# Several biological benefits of the low color temperature light-emitting diodes based normal indoor lighting source

**DOI:** 10.1038/s41598-019-43864-6

**Published:** 2019-05-17

**Authors:** Jiaqi Lin, Xingwei Ding, Can Hong, Yulian Pang, Liming Chen, Quanwen Liu, Xu Zhang, Hongbo Xin, Xiaolei Wang

**Affiliations:** 1 The National Engineering Research Center for Bioengineering Drugs and the Technologies, Institute of Translational Medicine, Nanchang University, Nanchang, Jiangxi 330031 China; 2 Affiliated Eye Hospital of Nanchang University, Jiangxi Research Institute of Ophthalmology & Visual Science, Nanchang, Jiangxi 330088 China

**Keywords:** Quality of life, Lasers, LEDs and light sources

## Abstract

Currently, light pollution has become a nonnegligible issue in our daily life. Artificial light sources with high color temperature were deem to be the major pollution source, which could induce several adverse effects on human’s health. In our previous research, we have firstly developed an artificial indoor light with low color temperature (1900 K). However, the biological effects of this artificial light on human’s health are unclear. Here, four artificial lights (1900 K, 3000 K, 4000 K and 6600 K) were used to evaluate some biological changes in both human (in total 152 person-times) and murine models. Compared with other three high color temperature artificial lights, our lights (1900 K) presented a positive effect on promoting the secreting of melatonin and glutamate, protecting human’s eyes, accelerating would healing and hair regeneration. These systematical studies indicated that the proposed low color temperature (1900 K) light could provide several significant benefits in human’s daily life.

## Introduction

**Figure 1:**
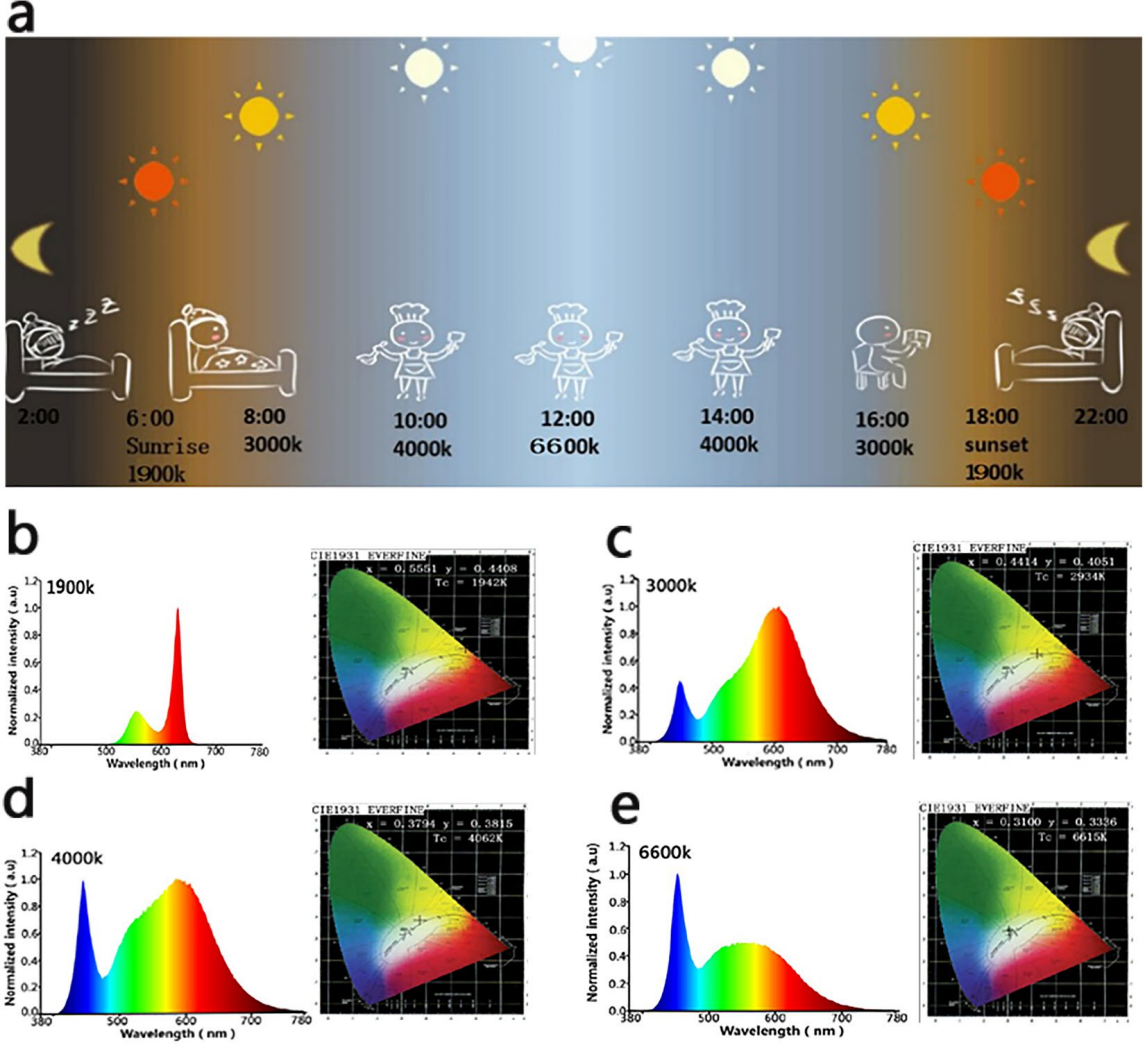
(**a**) Schematic illustration of the relationships between sun and human’s biological rhythm. (**b**–**e**) The electro-luminance spectra and CIE1931 chromaticity diagrams of 4 different color temperature lights (1900 K, 3000 K, 4000 K, and 6600 K)were characterized by integrating sphere. The electro-luminance spectra and CIE1931 chromaticity diagrams of four artificial lights with different color temperature (1900 K, 3000 K, 4000 K, and 6600 K) were characterized by integrating sphere.

Sun plays a significant role in human life for affecting human biological rhythms as the sole illumination since ancient times^1^. In China, there is an old saying called “rise with the lark and go to bed with the lamb” which is the interpretation about the relationships between sun and human biological rhythm. Generally, the color temperature of sun changes with time in the day, i.e. from sunrise (2000 K) to noon (6600 K), then to sunset (2000 K) in a sunny day^2^. With the changes of color temperature, human being instinctively modify their biological rhythm (sleep-work-sleep) (Fig. 1a). Therefore, this information indicated that the color temperature of sun is a remarkable factor for affecting human’s life^3^. Before the popularization of artificial lights in human’s life, firelight or candlelight (low color temperature) has been used for illumination with a history of 3.5 million years. With the development of human civilization, especially the advancement of light emitting diode (LED) technology, various artificial lights play an indispensable role in different areas of human life. However, due to the limitation of LED technology, current lights with low color temperature are difficult to achieve relatively high efficiency. As a result, the color temperature of illumination sources commonly used in human life is generally in the range of 3000 K–6600 K. As a comparison, the illumination sources with color temperature below 2000 K are relatively rare. Unfortunately, many studies have shown that the artificial lights with higher color temperature (3000 K–6600 K) interfered the normal biological rhythm of humans and even caused some diseases, such as insomnia, ophthalmopathy, depression, anxiety, inattention, gastrointestinal diseases, cardiovascular disease, breast cancer etc^4–8^. Therefore, it is reasonable to presume that artificial lights with low color temperature might compensate some of the above-mentioned defects, and provide certain benefits to human’s biological rhythm and health. Herein, the effects of artificial lights on human’s sleep, cognitive ability, eyes and wound healing were firstly assessed on volunteers treated with different artificial light (1900 K, 3000 K, 4000 K, 6600 K) respectively. Besides, we also made a tentative study on the wound healing and hair regeneration effects (through murine model) under the irradiation of different artificial lights. We hypothesized that the artificial light (1900 K), similar with sunrise and sunset, would supply some positive effects on human’s biological rhythm and health.

## Results and Disscusion

We used high-precision fast spectrum radiometer to characterize the physical property of four artificial lights. As shown in Fig. 1b, the spectra of light (1900 K) is concentrated from 500 nm to 650 nm, presenting yellow-orange-red light. The artificial lights (3000 K, 4000 K and 6600 K) presented a mixed light with the spectra ranging from 300 nm to 780 nm. It is worth noting that the blue light appeared in the lights with the color temperature of 3000 K, 4000 K and 6600 K, except 1900 K, which implied that the artificial light (1900 K) could reduce the adverse effect of blue light to eyes^9^. In addition, the color rendering index of light (1900 K) is 80 and the luminous efficiency is 66.73 lm/W, which indicated that it is an appropriate lighting source.

**Figure 2:**
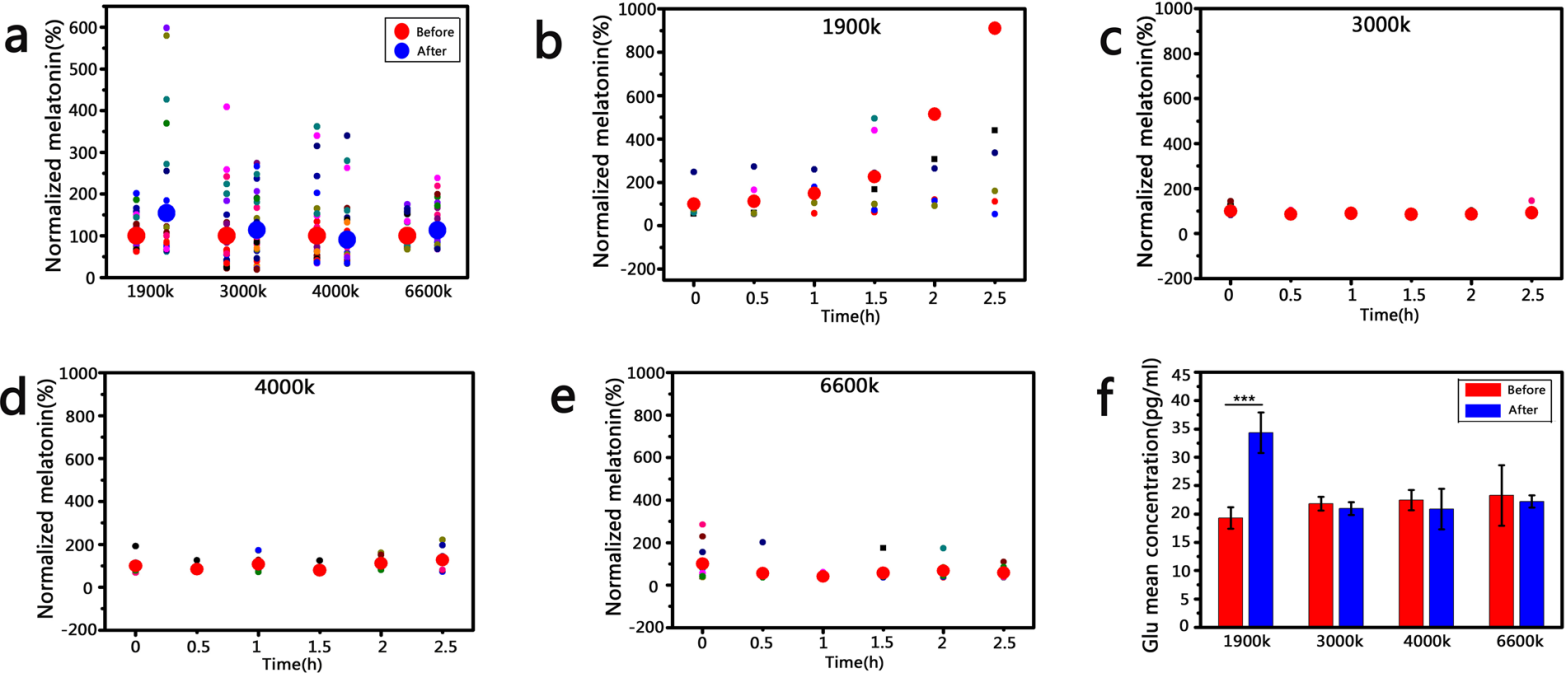
(**a**) The melatonin secretion from volunteers treated with artificial lights (1900 K, 3000 K, 4000 K and 6600 K) for 2 h (n ≥ 30). (**b**–**e**) The melatonin level of volunteers treated with artificial lights (1900 K, 3000 K, 4000 K and 6600 K) at the desired time (n ≥ 10). (**f**) The glutamate level of volunteers treated with artificial lights (1900 K, 3000 K, 4000 K and 6600 K) for 2 h (n ≥ 30) ***P < 0.001.

Melatonin, an important amino hormone, can improve the sleep quality^10–12^. To evaluate the biological effects of artificial lights on sleep, we monitored the melatonin level of 38 volunteers in all who were required to read under the irradiation of the four artificial lights (Fig. S1a,b), respectively. As shown in Fig. 2a, compared with the lights (3000 K, 4000 K and 6600 K), the average concentration of secreted melatonin is more than 1.5 times in the volunteers who treated with light (1900 K). Besides, we also found that the melatonin level increased by more than 400% in some individuals under the irradiation of light (1900 K), while the melatonin level of volunteers treated with other three light sources increased mildly (Fig. S2), which suggested that the light (1900 K) presented a better positive effect on sleep. To further assess the melatonin secreted behavior, the concentration of melatonin was monitored through measuring the collected saliva from volunteers for 2.5 h, the saliva was collected every 30 min. As shown in Fig. 2b, the results also suggested that the light (1900 K) could promote the secretion of melatonin, and we also found that the concentration of melatonin presented an obvious change after 2 h irradiation. The results showed that the secretion of melatonin presented a mild tendency before 1.5 h, but a sharp tendency after 2 h. It indicated that a large amount of melatonin was secreted after 1.5 h irradiation due to the biological response of human to yellow light. However, the concentration of melatonin changed mildly in volunteers who treated with light sources (3000 K, 4000 K and 6600 K), which indicated that these light sources supplied limited effect on melatonin secretion (Fig. 2c–e). These results provided a valuable reference for the practical application of light source with low color temperature (1900 K) in future.

With the bursting secretion of melatonin, human’s cognitive ability would be interfered due to the sleepiness. Glutamate secretion is a representative factor for characterizing human’s cognitive ability^13,14^. Therefore, the glutamate level was also measured through the collected saliva from volunteers treated with different light sources (1900 K, 3000 K, 4000 K and 6600 K) (Fig. 2f). Surprisingly, we found that only light source (1900 K) could also promote the glutamate secretion after 2 h irradiation. We then repeated the experiment twice to confirm this result. This information indicated that artificial light (1900 K) could not directly affect work efficiency. We supposed that the molecular mechanisms underlying the effect of light on melatonin and glutamate were different, resulting in the increased melatonin secretion with the accelerative glutamate secretion synchronously^11–15^.

Therefore, there are several advantages for the light source (1900 K): (1) The appropriate color rendering index (80%) make it to be an ideal normal light source for reading. (2) People could arrange their sleep time by pre-setting the light source (1900 K) irradiation to define the time of bursting secreted melatonin (about 2 h). (3) Before the sleep, the increased glutamate secretion is benefit to human’s cognitive ability.

**Figure 3:**
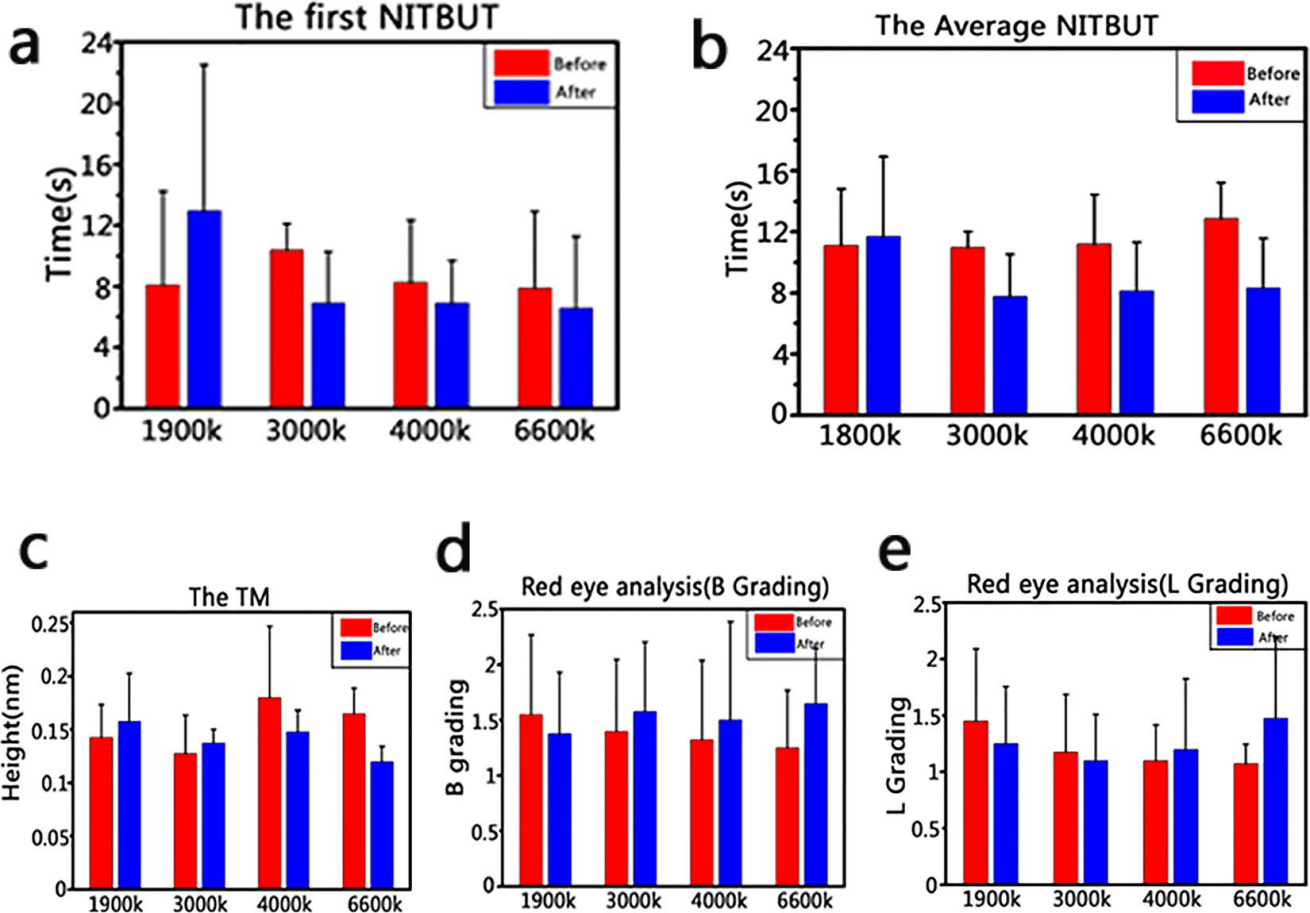
The biological effect on human’s eyes before and after irradiation by artificial lights (1900 K, 3000 K, 4000 K and 6600 K), the experiment was repeated by three times at different time. (**a**) The first non-invasive tear break-up time. (**b**) The average non-invasive tear break-up time. (**c**) The tear meniscus height. (**d**) The red eye analysis (B grading). (**e**) The red eye analysis (L grading).

On the other hand, the eyes are the direct organ to receive lights, and the images were formed in the brain through various processes. Therefore, to evaluate the effect of light on eyes, the non-invasive tear break-up time (NITBUT) and the average NITBUT were assessed through the same groups of volunteers after treating with different light sources (1900 K, 3000 K, 4000 K and 6600 K), respectively. As shown in Fig. 3a,b, the NITBUT was increased in the group of light source (1900 K), while NITBUT was decreased in the other three groups. It suggested that the stability of tear film was better in the group of light source (1900 K). Then we measured the tear meniscus height and red eyes analysis. The height of tear meniscus increased in the group of light sources (1900 K, 3000 K) (Fig. 3c) and the level of red eye was downgraded only in the group of light sources (1900 K) (Fig. 3d,e). These results also suggested that the light sources (1900 K) could relieve eye drying or red eye. We speculated that the wavelength of light sources (1900 K) was distributed near the red light and lacked the blue light, which made the light sources (1900 K) to be harmless to eyes.

**Figure 4:**
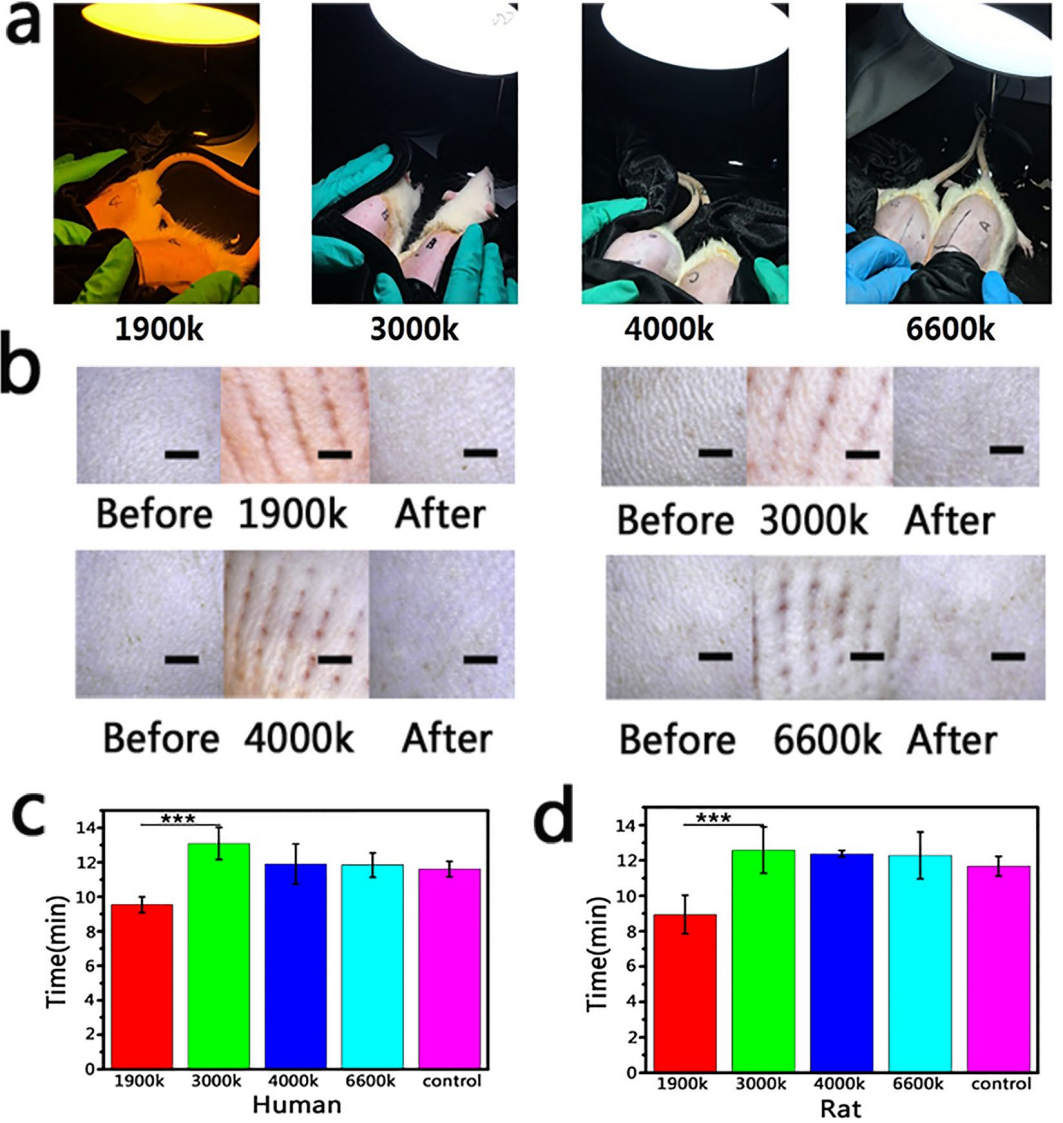
(**a**) The murine wound models were treated with different artificial lights (1900 K, 3000 K, 4000 K and 6600 K). (**b**) The human’s skin was irradiated by different artificial lights after being treated with microneedles (Scale bar: 20 μm). (**c**) The wound healing time of mice (n ≥ 3). (**d**) The wound healing time of human (n ≥ 4) ***P < 0.001.

**Figure 5:**
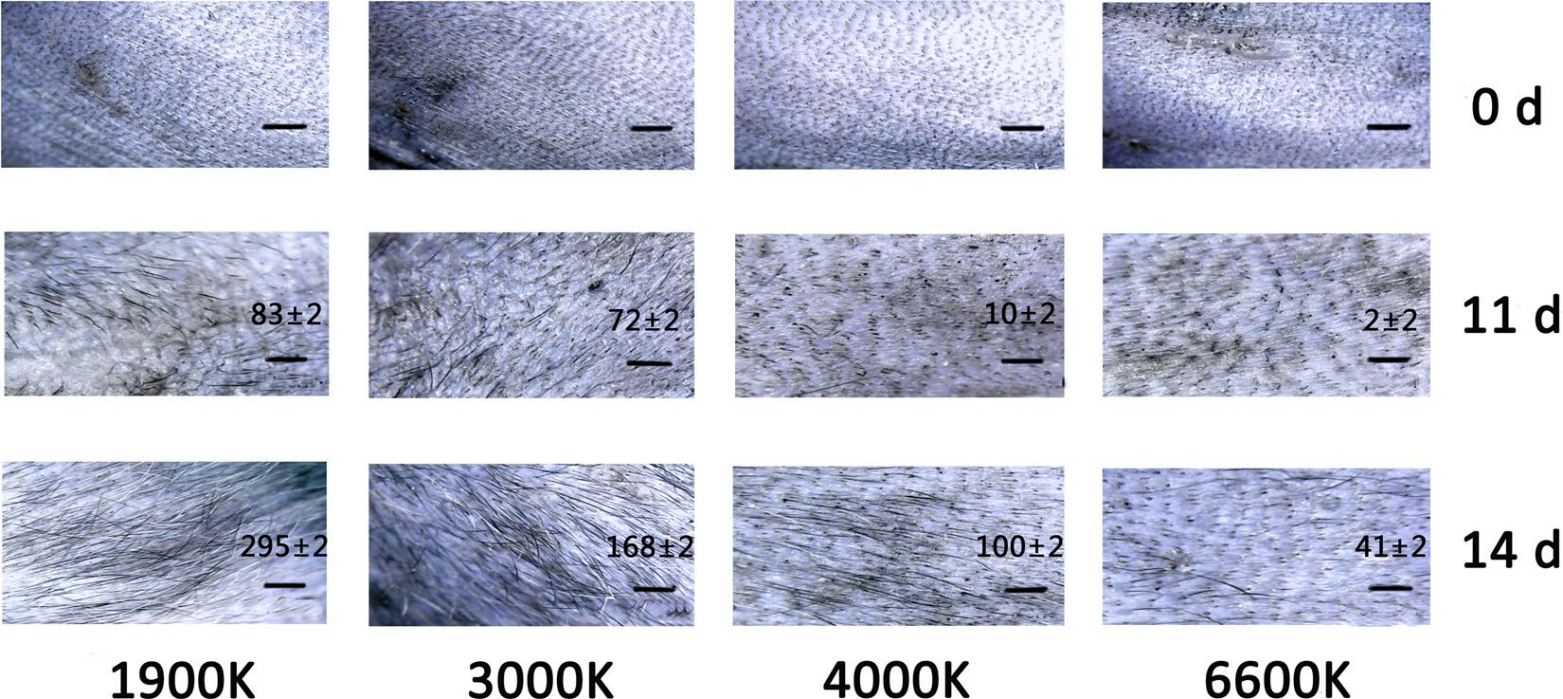
The hair regeneration assessment in murine alopecia model, the area of mice was irradiated by different artificial lights (1900 K, 3000 K, 4000 K and 6600 K) after being treated with stem cells growth factors (n ≥ 3) (Scale bar: 20 μm).

In the previous studies, red light was reported to be able to promote the wound healing and hair regeneration^16–19^. Besides, red light was used to promote tissue regeneration in burn and dermatology department^16,20^. In this study, the proposed light source (1900 K) has a large area of red light wavelength, so we hypothesized that light source (1900 K) also could promote wound healing and hair regeneration. Therefore, we evaluated the effect of light sources (1900 K, 3000 K, 4000 K and 6600 K) on wound healing (Fig. 4) and hair growth (Fig. 5) through building human and murine wound model and murine alopecia model respectively.

To build the wound model, microneedles were used to press on skin, then the volunteers or mice were irradiated under the different light sources, the healing time was recorded as the skin healing time (Fig. 4a,b). Significant difference was shown in the healing time of human and murine wound model between light source (1900 K) and other groups. The wound healing time presented less than 10 min in the group of light source (1900 K) only (Fig. 4c,d). The results suggested that light source (1900 K) could be used in our normal life to promote small area wound healing.

Red light was reported to be benefit to promote hair regeneration in the previous study^17–20^. Therefore, we speculated that the artificial light (1900 K) also could promote hair regeneration. As shown in Fig. 5, the mice were pre-treated by the equivalent amount of stem cells growth factors, which could stimulate hair regeneration^21,22^. Then the mice were irradiated by different light sources. After 2 weeks, the group of artificial light (1900 K) presented a faster hair growth speed than other three artificial lights (3000 K, 4000 K and 6600 K). It suggested that artificial light (1900 K) also could promote hair regeneration.

## Conclusion

In summary, the biological effect of light sources (1900 K, 3000 K, 4000 K and 6600 K) was systematically evaluated through assessing the melatonin level, the glutamate level, eye drying level, wound healing and hair regeneration. According to the results, light source (1900 K) presented several positive effects on human’s biological rhythm and health. Just like sunset, the light source (1900 K) is an “artificial light of harmony” which promote the secretion of melatonin, resulting in an improved sleeping quality. What’s more, because the large amounts of red light was included, it is also an “artificial light of life”, which could protect eyes and accelerate wound healing and hair growth. This promising indoor lighting source is thus expected to be applied in the field of advanced biomedical products such as sleep management, cosmetology and precise hair regeneration.

## Methods

### Materials

Artificial light (1900 K) was supplied by National Engineering Technology Research Center for LED on Silicon Substrate, Nanchang university; Artificial lights (3000 K, 4000 K and 6600 K) were purchased from Leishi illumination Co., Ltd. (Shenzhen, China); Salivary Melatonin ELISA Kit was purchased from Anbiqi Biotechnology Co., Ltd. (Shenzhen, China); Glutamate Saliva ELISA Kit was purchased from Jianglai Biotechnology Co., Ltd. (Jiangsu, China); Keratography 5 M was purchased from OCULUS Optikgeräte GmbH (Wetzlar, Germany) and schirmer strip was purchased from Jingming New Technological Development co., Ltd. (Tianjin, China). The depilatory cream was purchased from Yasheng Cosmetics Co., Ltd (Guangzhou, China) and the razor was purchased from Zhigao Air Conditioning Co., Ltd.(Guangzhou, China); The micro-needle wheel was purchased from Korea MTS Co., Ltd. Chloral hydrate, rosin, paraffin and cyclophosphamide were purchased from Macklin Co., Ltd. (Shanghai, China); The stem cell factor was supplied by Prof. Hongbo Xin and Dr. Quanwen Liu from Institute of Translational Medicine, Nanchang University.

### Characterization

The physical property of artificial lights was characterized by high-precision fast spectrum radiometer (Yuanfang Optoelectronic Information Co., Ltd., Hangzhou, China). The melatonin and glutamate level were measured by enzyme-linked immunosorbent assay (SpectraMax M5 type, Tianjin Science and Technology Development Co.). The digital microscope (Shenzhen Maimeiou Electronics Co., Ltd) was used to observe the skin and hair.

### Ethical approval

The ethical approval is obtained from the ethics committee of Nanchang University. All methods involving humans are performed in accordance with the relevant guidelines and regulations and informed consent was obtained from all participants. Informed consent for publication of identifying information/images in an online open-access publication was also obtained from all participants. All animal procedures are performed according to the protocol approved by the Institutional Animal Care and Use Committee at Institute (IACUC) of Translational Medicine, Nanchang University (Grant No. 2018NC-012-06), and the IACUC registration number of Institute of Translational Medicine of Nanchang University is SYXK 2015-0003.

### Melatonin and glutamate secretion assessment

Forty volunteers (18–25 ages) in all were invited to participate in the experiments about melatonin and glutamate secretion.

### Melatonin and glutamate secretion in 2 h

38 volunteers were invited to read in the dark environment only with the desired artificial light (Figs S1b,c and S3). The experiment started at 7:00 pm and lasted for 2 h. The volunteers were asked to collect 1 mL saliva at the start and end of the experiment. The collected saliva was centrifuged at 2000–3000 rpm for 15–20 min. Then, the supernatant was treated by salivary melatonin ELISA kit and glutamate saliva ELISA kit and the samples were measured by enzyme-linked immunosorbent assay at 450 nm.

### Melatonin secretion in 2.5 h

To evaluate the secreted melatonin more precise, 10 volunteers were invited to read in the dark environment only with the desired artificial light. The experiment started at 7:00 pm and lasted for 2.5 h. The saliva was collected from each volunteer every half an hour in the experiment. The collected saliva was centrifuged at 2000–3000 rpm for 15–20 min. Then, the supernatant was treated by salivary melatonin ELISA kit and the samples were measured by enzyme-linked immunosorbent assay at 450 nm.

### The biological effects assessment on eyes

4 volunteers in this experiment were chosen according to the below criteria: age ≥18 years; discontinuation of contact lens for more than 1 month; no history of family hereditary eye disease; no ocular trauma, history of laser and surgery; no signs of systemic immune disease and eye infection; no drugs affecting ocular surface function were used locally or in the whole body during two weeks; no abnormalities were observed in the slit lamp microscope and ophthalmoscopy; the best corrected visual acuity ≥1.0; the ocular surface disease index (OSDI) ≤ 12 points. The experiment was carried out in a dark room, volunteers were treated with the desired artificial light for 2 h.

### Non-invasive tear break -up time (NITBUT)

NITBUT measurements were got from the right eye by using the Keratograph 5 M. After manual focusing, we asked the volunteers to blink twice and then to keep their eyes open as long as possible. When there is irregular roundness or rupture roundness appeared in the Placido disk, the recording stops and the corresponding part is recorded as the first tear film break point. The first NITBUT was the first tear break-up time and the average value of the intervals after blinking across the observed area on the cornea were documented as the average NITBUT.

### Tear meniscus height (TMH)

The values of TMH were obtained on both eyes of volunteers using the Keratograph 5 M. Fixation targets in the Keratograph 5 M were a point in the center of the ring. Volunteers placed the head position in front of the central point of the head according to the inspection requirements, then were asked to look directly into the center of the ring, debugged the detector to the tear river height measurement mode, acquired images after the subject blinked, and measured the tear river below the center of the cornea using a random software caliper tool. Then, the Keratograph 5 M select the TMH setting, and the tear meniscus height could be measured.

### Measurement of conjunctival hyperemia (B grading)

This test was also used the Keratograph 5 M in the same physical space. We asked the volunteers to stare straight ahead and focus on the fixation target inside the Keratograph 5 M to allow the Placido ring system to be reflected in the entire corneal area. Then we clicked the capture button, there was a keratograph image created immediately and displayed on a computer screen. The system then was analyzed the image automatically and displayed a BR score (accurate to 0.1 unit) on the computer screen.

### Bulbar and limbal hyperemia (L grading)

We used the Keratograph 5 M to obtain the red eye index. Volunteers were demanded to keep their eyes open as wide as possible and focus on fixation target inside the Keratograph 5 M while the image was captured, then images were analyzed by the R-SCAN tool following the evaluation protocol software analyzed the image automatically and assigned a red eye index (accurate to 0.1 unit).

### Wound healing assessment

SD mice were obtained from Hunan SJA Laboratory Animal Co., Ltd. The mice were randomly divided into four groups (n ≥ 3). The hair was removed by depilatory cream and razor at the same area of rats’ back. Then, we used a microneedle roller to treat the rats’ back (Fig. S1d). After that, the mice were irradiated by different artificial lights in dark room. The wound healing was observed by digital microscope. The wound healing assessment also was evaluated on human. The arms of four volunteers were treated by microneedle roller, then the artificial lights were used to irradiate the treated area of arms. The wound healing was observed by digital microscope.

### The hair regeneration assessment

Male C57BL6 mice were obtained from Hunan SJA Laboratory Animal Co., Ltd. The murine alopecia model was prepared via the previous report. Briefly, mice were anesthetized by injecting with 2.5% chloral hydrate (0.01 mL/g) intraperitoneally. The melted rosin and paraffin were mixed in a ratio of 1:1, then the mixture was heated to 90 °C and smeared to the back of mice. After the mixture was solidified and hardened, the solidified mixture was removed and the depilatory area of each mouse was about 2.0 cm × 2.0 cm. After 7 days, mice were injected with cyclophosphamide (CTX) (150 mg/kg) subcutaneously in the depilatory areas. Then, the mice were randomly divided into four groups (n = 3), and 0.3 mL stem cell factor was smeared in the depilatory areas next day, following the irradiation of different lights respectively for 12 h in dark box. The experiment was carried out for 2 weeks and the hair of mice was monitored and record by a digital microscope every day.

### Statistical analysis

We used SPSS (IBM SPSS Statistics version 19) to analyze our experimental data, then analyzed our data for statistical significance with Student’s T-test and one-way analysis of variance (ANOVA).

## Supplementary information

Supporting info
