# Supplementary material for: Several biological benefits of the low color temperature light-emitting diodes based normal indoor lighting source
Source: Sci Rep. 2019 May 17;9:7560. doi: 10.1038/s41598-019-43864-6 (PMC6525242; doi:10.1038/s41598-019-43864-6)
Supplement: Supplementary file 1 — Supporting info [file 41598_2019_43864_MOESM1_ESM.pdf]

Supporting Information

Several biological benefits of the low color temperature light-emitting diodes based normal indoor lighting source

Jiaqi Lin[1,#], Xingwei Ding[1,#], Can Hong[1], Yulian Pang[2], Liming Chen[1], Quanwen Liu[1], Xu Zhang[2], Hongbo Xin[1], Xiaolei Wang[1,*]

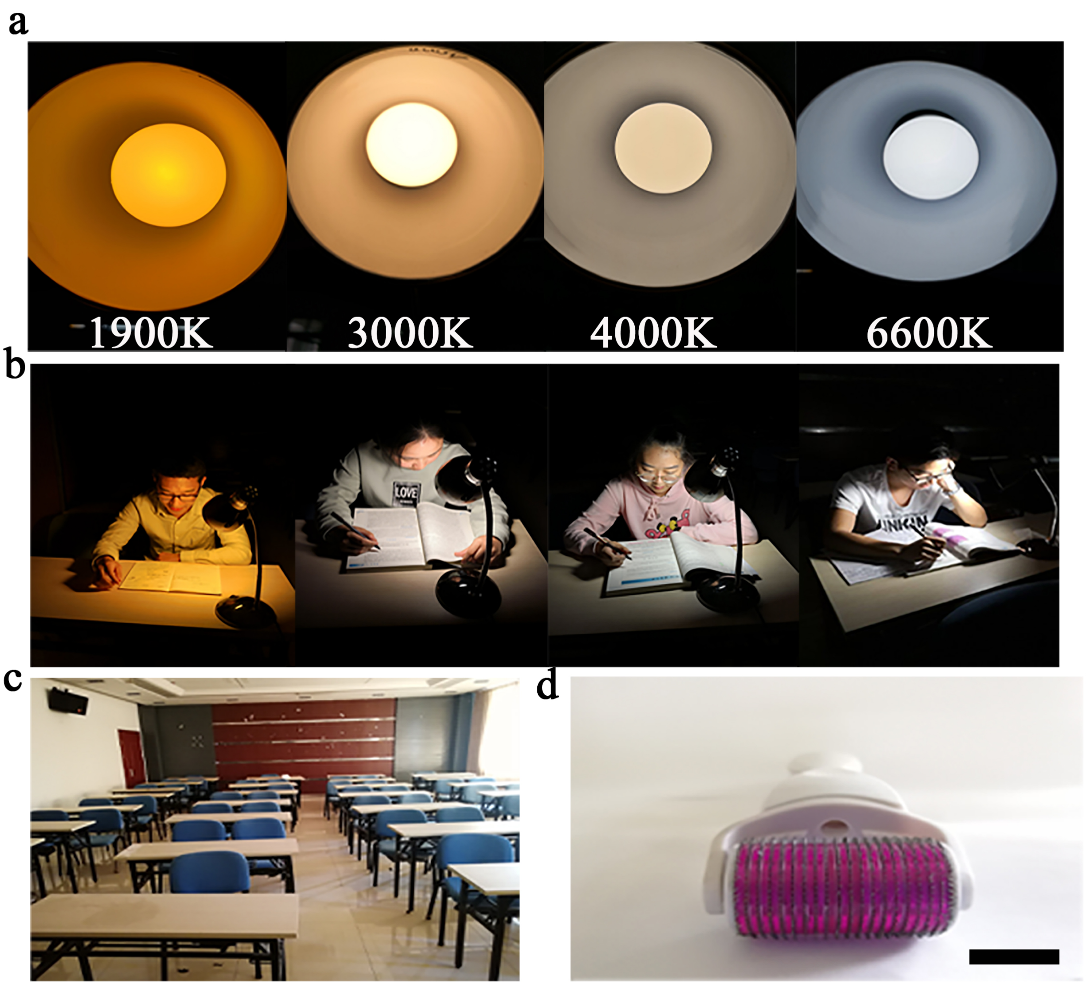

Figure S1: (a) Photos of four artificial lights. (b) Photos of volunteers who were reading

under the desired artificial lights in the dark room. (c) The dark room where the experiment was prepared. (d) Photos of the microneedle (Scale bar: 3 cm).

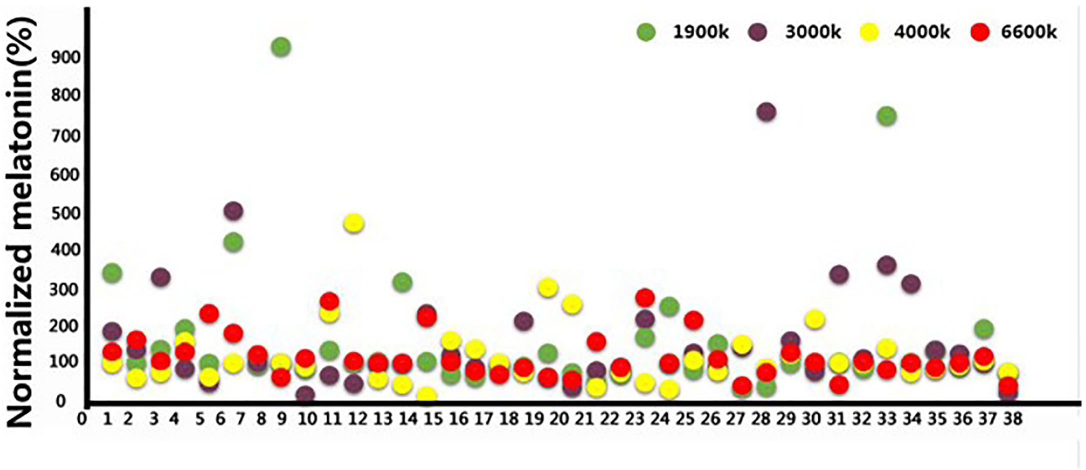

Figure S2: The melatonin secretion of 38 volunteers who were treated with four artificial lights for 2 h.

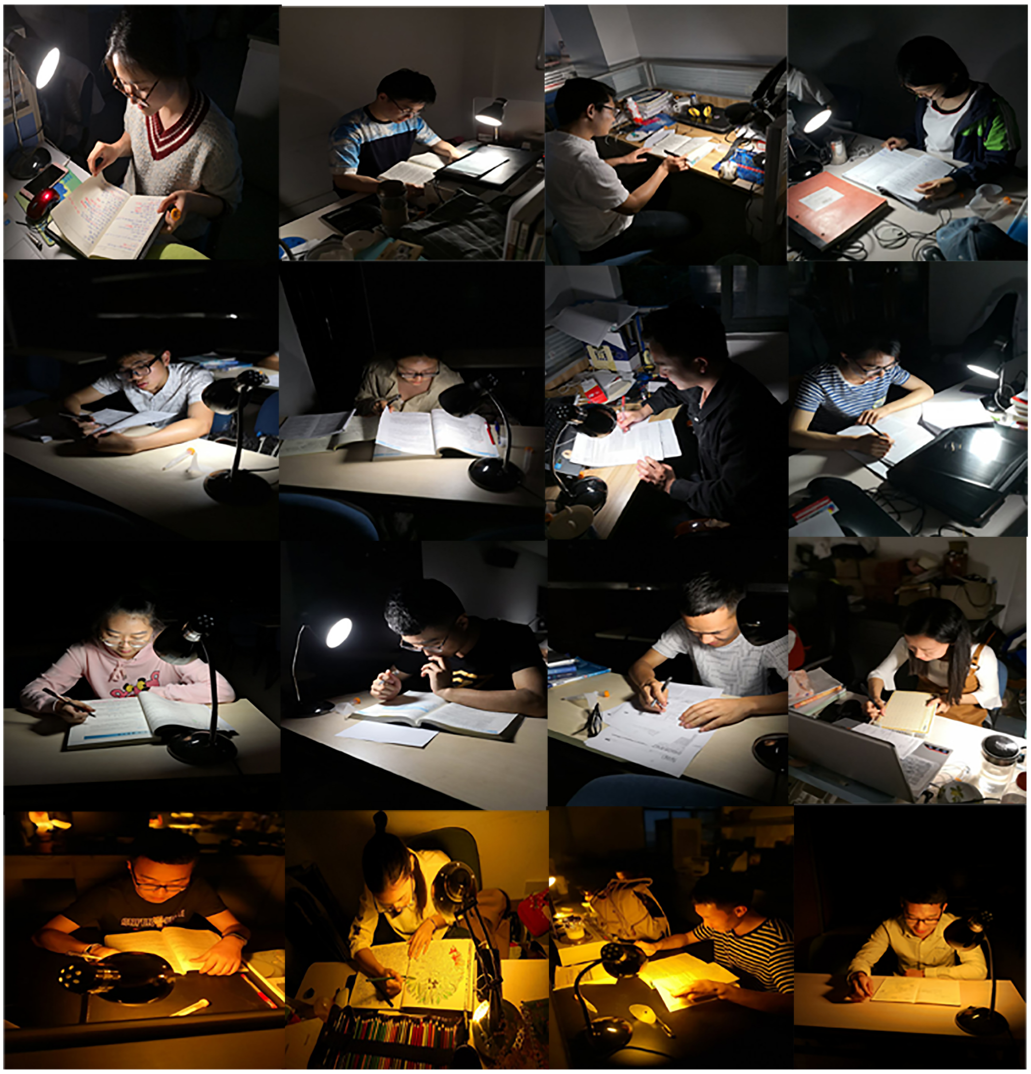

Figure S3: The photos of partial volunteers who were reading under the desired artificial lights.
